# Exploring the long-term use of ambroxol in Gaucher disease type 2: insights from two pediatric cases

**DOI:** 10.3389/fneur.2025.1690780

**Published:** 2026-01-23

**Authors:** Charlotte Aries, Anja Köhn, Karolin Täuber, Cornelia Rudolph, Tobias Böttcher, Peter Bauer, Steffen Fischer, Nicole Muschol

**Affiliations:** 1 International Center for Lysosomal Disorders, Department of Pediatrics, University Medical Center Hamburg-Eppendorf, Hamburg, Germany; 2 Centogene GmbH, Rostock, Germany

**Keywords:** enzyme replacement therapy, Gaucher disease type 2, high dose ambroxol, lysosomal storage disorder, neuropathic Gaucher disease

## Abstract

Gaucher disease 2 (GD2) is a rare and rapidly progressive neuropathic lysosomal storage disorder with an average survival of 11–19 months. To date, no approved therapy is available, but the variant-dependent pharmacological chaperone ambroxol (ABX) has emerged as a promising off-label therapy. This long-term observational study encompasses 2 GD2 patients treated with high-dose ABX from the age of 4 and 1 months, respectively, in addition to enzyme replacement therapy (ERT). Previously published data of patient 1 demonstrated a significant increase in β-glucocerebrosidase activity in ABX-treated patient fibroblasts alongside nearly age-appropriate neurocognitive and motor development after 3 years of ABX therapy. Follow-up assessments at the present age of 6.5 years continued to show normal neurocognitive development. Glucosylsphingosine (Lyso-GL1) levels in cerebrospinal fluid (CSF) remained significantly decreased compared to pre-treatment levels. In patient 2, ABX-treated fibroblasts exhibited a slight increase in β-glucocerebrosidase activity. Nevertheless, Lyso-GL1 levels in CSF showed a notable decrease compared to baseline. Neurocognitive and motor function assessments at 40 months of age indicated a moderate to severe developmental delay, yet continuous developmental progress. These interim findings contribute to the mounting evidence supporting ABX as a variant-dependent treatment for GD2 patients.

## 1 Introduction

Gaucher disease (GD) is a rare autosomal recessive lysosomal storage disorder caused by pathogenic variants in the *GBA1*-gene. The resulting deficiency of acid lysosomal β-glucocerebrosidase (GCase) leads to the accumulation of partially degraded substrates in macrophages, primarily glucosylceramide (GluCer) and glucosylsphingosine (Lyso-GL1) (1). This build-up of so-called Gaucher cells in the spleen, liver and bone marrow results in hepatosplenomegaly (HSM), bone lesions and pancytopenia (2). The disease has historically been classified based on the presence and severity of additional central nervous system (CNS) involvement but is increasingly perceived as a phenotypic continuum (3). The non-neuropathic form, type 1 (GD1), accounts for about 95% of GD cases and presents with only visceral symptoms. Neuropathic GD is a highly heterogenous disorder with a broad phenotypic spectrum and variability in onset and progression. The acute neuropathic type 2 (GD2) is associated with hydrops fetalis, congenital ichthyosis, abnormal muscle tone (hypo- or hypertonia), ophthalmoparesis, bulbar symptoms, failure to thrive, myoclonus, seizures and developmental delay (4, 5). A study analyzing 23 GD2 patients born since the year 2000 reported a mean age of death of 19 months in 20 deceased patients, with a range from 3–55 months (5). Type 3 (GD3) is considered the chronic neuropathic form, characterized by neurological manifestations such as impaired saccadic eye movements, myoclonic epilepsy and hydrocephalus, as well as systemic features including aortic calcification, interstitial lung disease and skeletal complications such as bone pain, osteopenia, kyphosis and scoliosis (4, 6).

Approved therapies exist only for GD1 and GD3 and comprise intravenously administered enzyme replacement therapy (ERT) and/or oral substrate reduction therapy (SRT). However, as these therapies do not cross the blood brain barrier, they do not address CNS involvement (6). Since its identification as a pharmacological chaperone for GCase in 2009, the secretolytic expectorant Ambroxol (ABX) has emerged as a promising off-label therapy for treating neuropathic GD (7, 8). By binding to mutant GCase in the endoplasmatic reticulum (ER), ABX promotes its folding and facilitates its transfer to the lysosome (9). The effectiveness varies depending on the specific *GBA1*-variant (10). In recent years, several case series, reports and reviews have demonstrated a favorable safety profile of high dose ABX treatment alongside with encouraging clinical and biochemical results. These include reduced ocular apraxia, dysarthria and stridor, improved ataxia, a decreased frequency of seizures, the achievement of developmental milestones and improved biomarkers (8, 11–16).

This long-term observational study presents 2 GD2 patients who received high dose ABX treatment from the ages of 4 months and 1 month, respectively, in combination with ERT. Data on patient 1 have previously been published following 3 years of ABX therapy, demonstrating an age-appropriate neurocognitive and motor development (17). The current analysis now includes an additional 3.5 years of clinical and biochemical follow-up confirming the sustained benefit of high dose ABX in combination with ERT on both neurological and visceral GD2 manifestations. These findings are further substantiated by data of patient 2, who received high dose ABX and ERT from 1 month of age and has now been in treatment for 3.5 years. These interim results contribute to the mounting evidence supporting the potential of ABX treatment as a variant-dependent treatment option for GD2 patients. Beyond adding longitudinal data, our work aims to illustrate the favorable clinical outcomes achievable with the combination of ABX and ERT and to provide practical guidance for clinicians navigating therapeutic decisions in this rare and heterogeneous disorder.

## 2 Materials and methods

### 2.1 Patient characteristics

The initial baseline characteristics of patient 1, along with data from the first 3 years of treatment, have already been published (17). Patient 1 is a female and the first child of consanguineous Turkish parents. Presenting with congenital ichthyosis at birth, diagnosis of GD2 was confirmed by reduced GCase activity and identification of the homozygous mutation p.R398L in the *GBA1*-gene. This mutation had previously been described only once in a Syrian boy who passed away at 18 months of age (18). Before initiation of ABX treatment at 3 months of age, the patient exhibited mild opisthotonos and hyperextension of the upper as well as lower limbs, moderate hyperexcitability and elevated muscle tone, as documented in the previously published video (link provided in the Supplementary material) (17).

Patient 2 is a female and the first child of non-consanguineous Albanian parents. She was born at term after an uneventful pregnancy and presented with jaundice at 2 days of age. Further investigations revealed hepatopathy and splenomegaly, necessitating transfer to our hospital. The diagnosis of GD2 was confirmed by reduced GCase activity and identification of the homozygous double-mutant allele p.[D409H;H294Q]. This is a common Albanian *GBA1*-allele associated with classical GD2 (19) or an intermediate phenotype between type 2 and type 3 (20).

### 2.2 Variant amenability assessment

The protocol of *in vitro* ABX treatment in patient-derived skin fibroblasts has already been published (17).

### 2.3 Treatment

ABX treatment was administered according to the pilot study on neuropathic GD by Narita et al. (12). Ambroxol hydrochloride (ambroxol ratiopharm solution, 7.5 mg/mL) was given at a dose of 25 mg per kg body weight per day, divided into three doses. As body weight of both patients increased, a higher-concentration formulation was prepared to ensure an appropriate dosing volume. Biweekly ERT with imiglucerase (60 IE per kg body weight, Cerezyme^®^, Sanofi-Aventis, Germany) was administered at the pediatric outpatient clinic.

### 2.4 Clinical and laboratory follow-up

Clinical follow-up data for patients 1 and 2 was collected during routine visits to the pediatric outpatient clinic. Basic physical examinations and assessment of adverse events were conducted at every biweekly visit, while detailed neurological examinations and blood tests were scheduled every 3 months.

Intervals for imaging and neurological assessments varied between the 2 patients, given that patient 1 had already been under treatment for 3 years, whereas patient 2 was treatment-naïve. Abdominal ultrasound imaging was scheduled at 6 months intervals for patient 1. In Patient 2, ultrasound examinations were initially performed monthly. After 4 months, the interval was extended to every 3 months. Patient 1 underwent follow-up cranial magnetic resonance imaging (cMRIs) at 3 years and again at 5 years and 4 months following the initiation of ABX treatment. Patient 2 received cMRIs at 17, 29 and 42 months of age.

Due to parental reports of episodes of altered awareness in patient 1, unscheduled follow-up electroencephalographies (EEGs) were performed 5 and 11 months after the previous investigation. Patient 2 underwent EEGs at diagnosis and then 4, 18 and 41 months after treatment initiation. Cerebrospinal fluid (CSF) opening pressure was measured once after 6 years and 4 months of ABX treatment for patient 1 and after 3.5 years of treatment for patient 2, prompting a follow-up measurement 3 months later. Ophthalmological examinations were scheduled on an annual basis.

### 2.5 Biochemical follow-up

Chitotriosidase and Lyso-GL1 in blood were analyzed every 3 to 6 months. Blood samples were collected in ethylenediaminetetraacetic acid (EDTA) tubes and analyzed via dried blood spots (DBS) for Lyso-GL1 and in plasma for chitotriosidase. CSF samples for Lyso-GL1 analysis were obtained annually for patient 1 and at baseline, after 4 months and then annually for patient 2. Lyso-GL1 levels were analyzed by Centogene (Rostock, Germany), while chitotriosidase levels were measured at the Biochemisches Labor (Villa Metabolica, Universitätsmedizin Mainz, Germany).

### 2.6 Developmental assessment

Neurocognitive and motor development were assessed annually using standardized tests appropriate for patients’ ages and abilities. Patient 1 was evaluated using the Kaufman Assessment Battery for Children II^®^ (KABC-II), while patient 2 was assessed using The Bayley Scales of Infant and Toddler Development^®^, Third Edition (BSID-III). Additionally, parental interviews using the Vineland Adaptive Behavior Scales^®^, Second Edition (VABS-II) were scheduled annually.

## 3 Results

### 3.1 Pre-treatment work-up

Patient 1 presented with muscular hypertonia and abnormal movement pattern prior to initiation of treatment at 4 months of age. Further details have been described previously (17).

Patient 2 exhibited opisthotonus as well as muscular hypotonia and HSM on clinical examination prior to initiation of treatment at 1 month of age. At birth, weight was at the 12th percentile and subsequently followed the 6–7th percentile trajectory. Abdominal ultrasound demonstrated HSM with markedly elevated elastography values (22 kPa—Ref. <5 kPa). Laboratory analyses revealed anemia (minimum hemoglobin 9.2 g/dL—Ref. 10.7–13.9 g/dL), thrombocytopenia (minimum platelet count 58*109/l—Ref. 200–460*109/l), elevated transaminases (maximum aspartate aminotransferase (AST) 953 U/L—Ref. 15–53 U/L; maximum alanine aminotransferase (ALT) 623 U/L—Ref. 5–39 U/L) and cholestasis (maximum gammaglutamyl transferase (GGT) 356 U/L—Ref. 5–17 U/L; maximum alkaline phosphatase (AP) 594 U/L—Ref. 97–136 U/L). Due to the hepatopathy, the diet was changed to specialized formula intended for infants with liver disease. Metabolic testing demonstrated low GCase activity of 32.83 pmol/spot*20 h (Ref. 200–2,000 pmol/spot*20 h) in DBS and increased blood biomarkers (chitotriosidase 3,173 nmoL/mL/h—Ref. 20–100 nmoL/mL/h; Lyso-GL1 1,430 ng/mL—Ref. <6.8 ng/mL). LysoGL1 in CSF was markedly elevated (2,340 pg/mL—Ref. 1.5–38.7 pg/mL).

### 3.2 Variant amenability assessment

**Figure 1:**
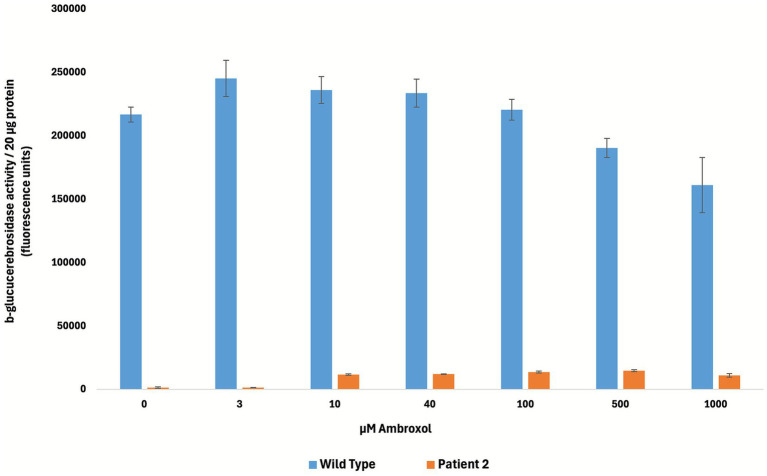
Chaperone effect of ABX on GCase activity in cultured fibroblasts from patient 2 compared to healthy donor cells, demonstrating a noticeable increase in GCase activity in patient fibroblasts at concentrations of 10 μM and above.

*In vitro* experiments in fibroblasts of patient 2 isolated prior to ABX treatment demonstrated an increase in GCase activity up to 5% of wild type activity (Figure 1), compared to 57% in patient 1 as previously reported (17).

### 3.3 Treatment

As previously reported, patient 1 received ABX from 4 months of age. Due to an insufficient response in visceral disease manifestations, ERT was added to the treatment regimen at 15 months of age (17). Patient 2 was started on ABX at 1 month of age. ERT was added 2 weeks later. The combination therapy was well tolerated by both patients.

### 3.4 Clinical and laboratory follow-up

Patient 1 demonstrated stable hematological parameters within the normal range. Transaminase levels remained within normal limits, whereas GGT persistently showed a mild elevation, ranging from 19–55 U/L (Ref. 9–20 U/L). Three years after initiation of ABX treatment (and 2 years after start of ERT), hepatic ultrasound imaging revealed heterogenous parenchyma and elevated elastography values (11.8 kPa). While the heterogenous appearance of the liver remained unchanged over the subsequent 3.5 years, elastography values increased to 14.8 kPa a year later and remained stable over the following 2.5 years. Liver size decreased over time, reaching normal values after 5 years and 4 months of ABX treatment and 4 years and 4 months following the initiation of ERT. Mild splenomegaly persisted throughout the observation period. Discrete parenchymal irregularities were first identified 4 years and 2 months after treatment initiation with ABX and have shown no progression to date. Regular neurological examinations and follow-up cMRIs yielded no pathological findings. A single CSF opening pressure measurement obtained 6 years and 4 months after initiation of ABX treatment was within the normal range (12 cmH_2_O). At the age of 2 years and 3 months, the parents reported episodes of altered awareness during play or video watching. However, a long-term EEG and a follow-up EEG 6 months later revealed no abnormalities. Ophthalmological evaluation showed peripheral retinal thinning on optical coherence tomography (OCT), which remained stable over time.

Patient 2 demonstrated normalization of hemoglobin 3 days after initiation of ABX treatment. Platelet counts normalized 7 weeks after the initiation of ABX treatment and 5 weeks after the initiation of ERT. AST levels gradually declined, reaching the normal range after 8 months. ALT levels followed a more fluctuating course but showed an overall downward trend, normalizing after approximately 3 years. GGT levels, while generally decreasing as well, have remained slightly elevated to date (25 U/L—Ref. 5–17 U/L). Hepatomegaly persisted during the first 2.5 years of treatment, after which liver size remained at the upper limit of normal. Elastography values showed an initial pronounced decline in follow-up measurements at 7 and 11 weeks after initiation of ABX and 5 and 9 weeks after the start of ERT, reaching 14.7 kPa and 9.9 kPa at the respective time points. Subsequent assessments demonstrated moderate fluctuations with an overall downward trend. At the most recent evaluation, 3 years and 3 months after treatment initiation, elastography values had just reached the upper limit of normal.

Up to 2 years and 4 months into treatment, the hepatic parenchyma was described as mildly irregular. Splenomegaly persisted throughout the observation period but had resolved by the most recent assessment, conducted 3 years and 4 months after treatment initiation. Neurological examination revealed a reduction in opisthotonus within a few months after initiation of therapy. Over time, an eye movement disorder characterized by impaired vertical saccades became evident. Ophthalmological evaluation revealed oculomotor apraxia. Detailed assessment of this condition was complicated by the development of a pronounced cervical spine retroflexion secondary to kyphosis. While a cMRI scan at 17 months of age showed no abnormalities, imaging conducted at 29 months of age revealed enlarged ventricles. A follow-up cMRI at 42 months of age demonstrated increasing enlargement of the ventricles, which correlated with an elevated CSF opening pressure (44 cmH_2_O). No prior measurements of CSF opening pressure had been performed. After interdisciplinary consultation involving pediatric neurologists and neurosurgeons, acetazolamide therapy was initiated. A follow-up measurement at 45 months of age revealed a reduction in CSF opening pressure to 26 cmH_2_O. Regular EEGs showed no clinically significant abnormalities.

### 3.5 Biochemical follow-up

**Figure 2:**
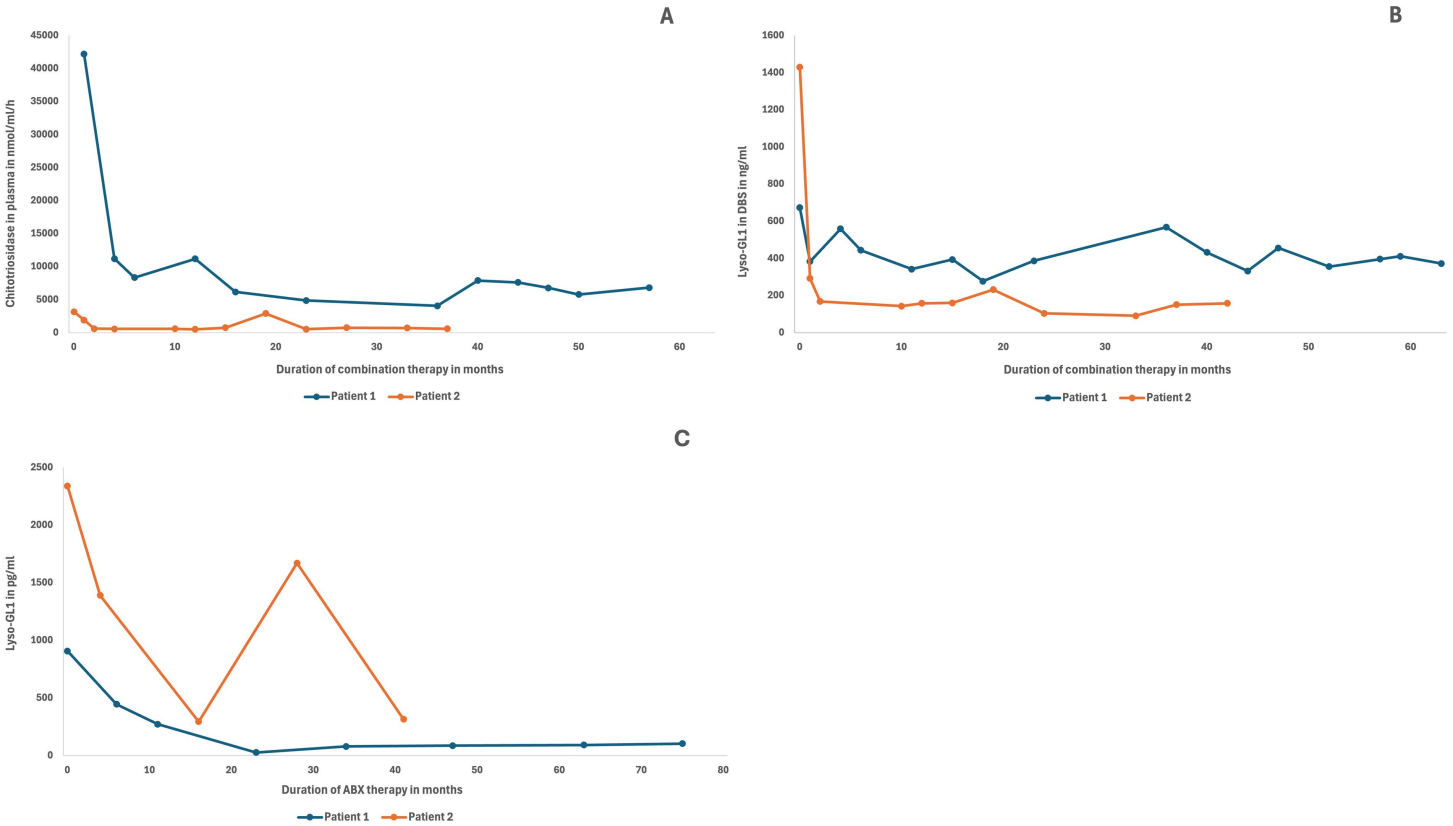
Chitotriosidase in plasma, Lyso-GL1 in DBS, and Lyso-GL1 in CSF. Prompt decrease and subsequent stabilization of chitotriosidase in plasma **(A)** and Lyso-GL1 in DBS **(B)** after initiation of combination therapy in both patients. Prompt decrease of Lyso-GL1 in CSF **(C)** after initiation of ABX therapy in both patients, subsequent stabilization in patient 1, and interim rebound due to compliance issues in patient 2.

Patient 1 showed an initial reduction, followed by stabilization of plasma chitotriosidase and Lyso-GL1 levels in blood after initiation of combination therapy (Figure 2). During ABX monotherapy, both biomarkers had exhibited an increasing trend (17). Lyso-GL1 in CSF remained stable throughout the treatment period, having markedly decreased by 97% after 24 months of ABX treatment as previously reported (17).

Patient 2 showed a rapid decline of biomarkers in blood (chitotriosidase and Lyso-GL1) (Figure 2). LysoGL1 in CSF initially decreased by 41% to 1,390 pg/mL after 4 months of treatment and further declined to 275 pg/mL after 16 months, reflecting an overall 88% reduction from baseline. At 28 months after treatment initiation, there was a rebound up to 1,670 pg/mL due to suboptimal adherence. The most current control at 41 months showed another decrease down to 316 pg/mL.

### 3.6 Developmental assessment

Neurocognitive testing with the KABC-II indicated age-appropriate development for patient 1 at ages 4 years and 4 months, 5 years and 7 months and 6 years and 7 months with nonverbal index (NVI) scores of 92, 97 and 97, respectively (Table 1). In the VABS-II, patient 1 demonstrated a “medium low” adaptive level at the first two of these time points, indicating mildly reduced adaptive functioning relative to age norms. Video documentation displayed age-appropriate development at 6 years and 7 months (Supplementary material).

**Table 1:** Neurocognitive and motor development assessments.

A	Patient 1: Kaufman Assessment Battery for Children-II® (KABC-II)			
		Age		
		4 y 4 m	5 y 7 m	6 y 7 m
	Nonverbal index	92 (84–108)	97 (90–105)	97 (90–104)

B	Patient 1: Vineland Adaptive Behavior Scales®, Second Edition (VABS-II)		
	Domain	AEqs at age of	
		4 y 4 m	5 y 7 m
	Communication	2 y 3 m	2 y 6 m
	Daily life skills	3 y 7 m	3 y 4 m
	Social	3 y 3 m	3 y 3 m
	Motor	3 y 7 m	3 y 10 m
	Standard score	84 (81–87)	72 (67–77)
	Adaptive level	Medium low	Medium low

C	Patient 2: Bayley Scales of Infant and Toddler Development®, Third Edition (BSID-III)				
	Domain	AEqs at age of			
		12 m	19 m	32 m	40 m
	Cognitive development	9 m	11 m	21 m	24 m
	Receptive language	4 m	n.d.	n.d.	20 m
	Expressive language	8 m	n.d.	n.d.	n.d.
	Fine motor	11 m	n.d.	24 m	27 m
	Gross motor	9 m	n.d.	n.d.	n.d.

D	Patient 2: Vineland Adaptive Behavior Scales®, Second Edition (VABS-II)			
	Domain	AEqs at age of		
		12 m	32 m	42 m
	Communication	9 m	18 m	23 m
	Daily life skills	10 m	17 m	45 m
	Social	8 m	19 m	25 m
	Motor	11 m	19 m	23 m
	Standard score	82 (78–86)	72 (68–76)	76 (71–81)
	Adaptive level	Medium low	Medium low	Medium low

AEqs, age equivalents; y, years; m, months; n.d., not done due to lack of cooperation.

Patient 2 was evaluated with the BSID-III at 12, 19, 32 and 40 months of age (Table 1). At 12 months, the developmental quotient (DQ) was 75, reflecting a moderate developmental delay. Subsequent assessments indicated a more pronounced developmental delay with DQ scores of 58 at 19 months, 66 at 32 months and 59 at 40 months (Table 1). Nevertheless, continuous developmental progress was observed. In the VABS-II, patient 2 showed a “medium low” adaptive level at 12, 32 and 42 months, consistent with mildly reduced age-appropriate adaptive functioning. Video documentation at 3 years and 5 months demonstrated patient 2 walking independently, following instructions and drawing, while simultaneously exhibiting the marked cervical spine retroflexion (Supplementary material).

## 4 Discussion

GD2 is a severe and rapidly progressive lysosomal storage disorder, characterized by the onset of neurovisceral symptoms in infancy and typically resulting in death during early childhood (5). Currently, no approved therapies target the CNS manifestations of the disease. However, the variant-dependent pharmacological chaperone ABX has emerged as a promising off-label treatment, frequently used as an add-on to ERT (8).

Patient 1 showed a sustained and remarkable treatment response to long-term therapy with ABX and ERT. Neurological assessments and cMRI demonstrated no abnormalities. Neurocognitive and motor development remained age-appropriate and Lyso-GL1 levels in CSF were stable. These findings extend observations from a recent review analyzing 21 studies on ABX treatment for GD, with follow-up periods ranging from 6 months to 7 years (8). The review reports various clinical benefits such as improved laboratory parameters, reduced HSM, survival without tracheostomy and decreased severity of neurological symptoms including seizures, ocular apraxia, dysarthria and stridor. However, treatment responses varied widely. Notably, among all included GD2 cases, only the patient presented here (patient 1) was reported to have achieved age-appropriate developmental milestones by 3 years of age. The present long-term follow-up demonstrating continued age-appropriate development at 6.5 years further highlights both the potential of ABX as a variant-dependent therapeutic approach in selected patients and the critical importance of early intervention.

As hepatic ultrasound revealed heterogenous parenchyma and persistently elevated elastography values over the last 3.5 years, the option of a liver biopsy is currently being discussed with pediatric hepatologists. Liver involvement beyond hepatomegaly, such as focal liver lesions and fibrosis with an associated increased risk of hepatocellular carcinoma (HCC), is a well-established feature of both non-neuropathic and neuropathic GD (28). While hepatomegaly and fibrosis generally improve with ERT, focal liver lesions often persist and the prevalence of steatosis increases with longer treatment duration (21). Close clinical monitoring is therefore essential and further diagnostic evaluation should be considered in cases of clinical uncertainty.

Patient 2 presented with hepatopathy shortly after birth. Genetic analysis identified homozygosity for the double mutant allele p.[D409H;H294Q], a common *GBA1*-variant in the Albanian population (22). This genotype has previously been reported in 3 patients with classical GD2 (19) and in 2 patients with an intermediate phenotype between GD2 and GD3 (20, 23). While the first 3 patients were only briefly described as exhibiting a very severe early-onset neurological phenotype, the latter 2 cases were characterized in more detail. Both patients presented with neck rigidity, head retroflexion and oculomotor apraxia at 5 and 6 months of age, respectively. One of them remained neurologically stable at the age of 3 years with improved visceral manifestations under ERT (20). The other showed slowly progressing neurological symptoms, but was able to sit unaided, walk with support and speak simple words by 25 months of age, with improvement of visceral manifestations after initiation of ERT at 13 months of age. Brainstem evoked potentials were abnormal and cMRI revealed abnormalities in the pons, cerebellum, periventricular areas and basal ganglia (23). The patient died at the age of 3 years (22).

Combination therapy with ABX and ERT in patient 2, initiated at 1 month of age, led to a reduction in HSM, plasma chitotriosidase and Lyso-GL in both blood and CSF. *In vitro* testing in patient-derived fibroblasts demonstrated a slight dose-dependent increase in GCase activity, reaching up to 5% of wild-type activity.

On neurological examination, the patient exhibited impaired vertical eye saccades, oculomotor apraxia and a marked retroflexion of the cervical spine attributable to kyphosis. The cMRIs demonstrated progressive hydrocephalus. Elevated CSF opening pressure led to the initiation of acetazolamide treatment. The *GBA1*-variant p.[D409H] has previously been associated with communicating hydrocephalus, potentially caused by leptomeningeal fibrosis or thickening of arachnoid villi, which may impair CSF distribution and absorption (24, 25). Neurocognitive testing of patient 2 demonstrated an initially moderate developmental delay, which became more pronounced over time. As in patient 1 at a comparable age, assessment of speech development presented a challenge due to bilingualism, with Albanian as the currently dominant language. Nevertheless, the patient continued to make developmental progress. An intermittent rebound in Lyso-GL1 levels in CSF was attributed to suboptimal adherence. Following correction, Lyso-GL1 levels declined again, highlighting the substantial impact of ABX on GCase activity and the importance of patient adherence for maintaining therapeutic benefit.

When following patients on experimental treatment, it is essential to continuously assess therapeutic efficacy and weigh benefits against potential drawbacks. This requires consideration of the disease’s natural history in the absence of such interventions. A recent proposal for a descriptive classification of GD2 patients, aiming to better capture their heterogeneity and to facilitate more accurate prognostic assessment, distinguishes five clinical categories (26). Applying this framework to our patients at the time of diagnosis, patient 1 cannot be unambiguously assigned to a single category, as she meets isolated criteria from several groups. She presented with a collodion baby phenotype at birth, a feature included in both the “Gaucher-related hydrops” and the “neonatal inflammatory” categories. She also had elevated inflammatory markers during the first months of life (ferritin 716 μg/L—Ref. 9–159 μg/L), though approximately 10-fold lower than in patient 2, fulfilling another criterion of the “neonatal inflammatory” category. In addition, increased muscle tone and hyperextension of the limbs were first documented at 3 months of age (exact time of onset unclear), which is later than 1 month but before 6 months of age and corresponds to features described in the “early infantile neurodegenerative” category. However, her partial concordance with several of these categories may further reflect the wide phenotypic spectrum of neuropathic GD, and the nature and time of onset of her symptoms consistently support her classification as GD2 (27). Patient 2, in comparison, clearly aligns with the “neonatal inflammatory” category, characterized by hepatic involvement, thrombocytopenia and markedly elevated inflammatory markers (ferritin 7653.5 μg/L—Ref. 9–159 μg/L) within the first weeks of life. Given their classification as GD2, their developmental trajectories deviate markedly from the expected course. Patient 1 remains a uniquely successful case (8). Nonetheless, patient 2 has also reached significant developmental milestones. Abilities like communicating in multi-word sentences and walking independently clearly distinguish her from previously described GD2 patients (5). Despite their clinical differences and marked differences in *in vitro* responses to ABX (GCase activity in patient fibroblasts of 57% vs. 5% of wild-type activity in patients 1 and 2, respectively), both cases illustrate the potential of ABX to target CNS disease manifestations (17). Regarding safety, ABX has been well tolerated by both patients. Patient 1 experienced transient increased mucus production during the first weeks of treatment, which led her parents to administer the syrup more slowly. She has had no further side effects. Patient 2 has not exhibited any side effects.

In conclusion, ABX continues to demonstrate considerable clinical benefit alongside a favorable safety profile, highlighting its potential as a variant-dependent therapeutic option for GD2. However, navigating a diagnosis of GD2 and making decisions regarding the scope and goals of treatment remains a sensitive and challenging issue (26). ERT monotherapy may prolong life and improve visceral and hematological disease manifestations, but it does not prevent neurological progression and may prolong the overall disease course, raising legitimate ethical concerns (5). Experimental add-on therapies aimed at addressing neurological symptoms, such as variant-dependent ABX, may provide clinically meaningful effects in selected patients. In light of the limited therapeutic options currently available, we suggest prompt initiation of ABX therapy in combination with ERT upon diagnosis, in parallel with an amenability assessment using patient derived fibroblasts. Alternatively or in addition, measurement of Lyso-GL1 in CSF may be considered. Parents should receive empathetic support with comprehensive and transparent counseling on the potential benefits and limitations of treatment, including their experimental nature. Treatment continuation should be guided by the individual biochemical and clinical response over time.

## Data Availability

The datasets presented in this article are not readily available because of ethical and privacy restrictions. Requests to access the datasets should be directed to the corresponding author.

## Glossary

ABX: Ambroxol
ALT: Alanine aminotransferase
AP: Alkaline phosphatase
AST: Aspartate aminotransferase
cMRI: Cranial magnetic resonance imaging
CNS: Central nervous system
CSF: Cerebrospinal fluid
DBS: Dried blood spots
DQ: Developmental quotient
EDTA: Ethylenediaminetetraacetic acid
EEG: Electroencephalography
ER: Endoplasmatic reticulum
ERT: Enzyme replacement therapy
GCase: β-glucocerebrosidase
GD: Gaucher disease
GGT: Gammaglutamyl transferase
GluCer: Glucosylceramide
HCC: Hepatocellular carcinoma
HSM: Hepatosplenomegaly
Lyso-GL1: Glucosylsphingosine
OCT: Optical coherence tomography
SRT: Substrate reduction therapy
