# Supplementary material for: Exploring the long-term use of ambroxol in Gaucher disease type 2: insights from two pediatric cases
Source: Front Neurol. 2026 Jan 23;16:1690780. doi: 10.3389/fneur.2025.1690780 (PMC12877786; doi:10.3389/fneur.2025.1690780)
Supplement: Supplementary file 1 [file Data_Sheet_1.docx]

**Supplementary material**

The video is archived on Zenodo and can be accessed here:

<https://zenodo.org/records/15839224?token=eyJhbGciOiJIUzUxMiJ9.eyJpZCI6IjUwYmU0ZDhlLTI3MjAtNGNhZS05MDhiLWM0MTUwMWE2NTQ3NyIsImRhdGEiOnt9LCJyYW5kb20iOiI0OWNjOWU5YmRkNzc0MTIzMDk3YmMzZWQ3Y2ZlYjY2ZSJ9.-zj9TqCeCeFK0LM1jlUOU15Wci5gzt_WeTw4wSHb7AKdH32cwu7ouPPemptGIlVbHk4MHu0gMj02yRheNb46lA>
